# Cerebral and Spinal Cord Protection Strategies in Aortic Arch Surgery

**DOI:** 10.3390/jcdd12040130

**Published:** 2025-04-02

**Authors:** Andrea Myers, Ciprian Nita, Guillermo Martinez

**Affiliations:** Royal Papworth Hospital Foundation Trust, Cambridge CB2 0AY, UK

**Keywords:** aortic arch surgery, frozen elephant trunk, neuroprotective strategies, spinal cord protection, hypothermic circulatory arrest, perfusion

## Abstract

Perioperative management of patients undergoing surgeries of the aortic arch is challenging. This cohort of patients has a high risk of poor neurological outcomes both as a consequence of the disease process as well as the methods employed during surgical management. Many strategies have been put forward to ameliorate these complications; however, maintaining cerebral and spinal cord perfusion and reducing metabolic oxygen demand is the core principle of these strategies. Moderate hypothermia and selective ante-grade perfusion are the most promising methods that provide the best conditions for the competing requirements of both the brain and spinal cord. Intraoperative and postoperative monitoring is essential for early detection and intervention in delayed spinal cord ischaemia and stroke. In this article we aim to discuss the current methods of neuroprotection and spinal cord protection in aortic arch surgery and stenting.

## 1. Introduction

Aortic arch replacement surgery is one of the most complex procedures of the aorta, with an in-hospital mortality rate above 10% [1]. Surgical repair of the aortic arch requires periods of interruption of the aortic blood flow to the brain and lower body, which can lead to irreversible nervous system damage [2]. Over the past decades, advances in surgical techniques that maximise organ perfusion have improved perioperative outcomes [3,4]. This article intends to explore the current evidence on strategies that enhance end-organ protection and contribute to reducing the catastrophic complications associated with aortic arch surgery, such as stroke and paraplegia.

## 2. Cerebral Neuroprotection for Aortic Arch Surgery

Implementing neuroprotective strategies during aortic arch surgery is essential for (the best) optimal neurological outcomes. Strategies to measure and maintain perfusion, targeted temperature management and pharmacological interventions are the core therapies to prevent perioperative stroke, spinal cord injury and other neurological complications [2].

Deep hypothermic circulatory arrest (DHCA) has been the classic strategy for performing aortic arch surgery [5]. During DHCA (14.1 to 20.0 degrees Celsius), all organ perfusion stops for a period between 20 and 40 min and then restarts once the vascular anastomosis is completed [6]. Although most centres practise DHCA, the ideal temperature for cerebral protection is unknown. DHCA has been associated with cerebral oedema and reperfusion injury which manifest as postoperative cognitive dysfunction and stroke [7]. Current practise advocates the use of moderate hypothermic circulatory arrest (MHCA) (25–28 degrees Celsius) to reduce the complications associated with DHCA as studies have shown it to be non-inferior to DHCA for cerebral protection [7]. However, maintaining either antegrade or retrograde cerebral perfusion has shown better neurological outcomes than DHCA or MHCA alone [8]. Selective antegrade cerebral perfusion (SACP) can be delivered using one or more cannulas to provide blood flow to the brain [9]. Using unilateral axillary cannulation for antegrade perfusion allows the maintenance of flow in the aortic arch, great vessels and descending aorta during the cooling period on cardiopulmonary bypass (CPB). It also offers adequate SACP during lower-body arrest, and it has shown a reduced risk of embolisation of atheromatous plaque, aortic rupture, false lumen perfusion and perioperative mortality compared to aortic and femoral cannulation [10,11]. Adequate oxygen delivery in the brain can be measured using near-infrared spectrometry (NIRS) that estimates the regional haemoglobin saturation of the brain (rSO_2_). NIRS can measure rSO_2_ up to 3–4 cm in cortical depth but are limited to the frontal lobes. Since up to 20 to 30% of the population has an incomplete Circle of Willis with a lack of connection between the anterior and posterior circulation, a unilateral axillary SACP may not always be sufficient to perfuse the contralateral and posterior cerebral compartments [12,13]. Limited anatomical studies suggest that blood flow via the ipsilateral vertebral artery, leptomeninges and collateral circulation may compensate for gaps in the Circle of Willis. Although various methods of arch cannulation have been trialled to improve cerebral blood flow, these have not shown improved mortality benefit compared to SACP [14]. This may be due to the increased risk of micro-emboli from multiple cannulation sites. Measuring anterior rSO_2_ does not capture deep and posterior compartments, and ensuring the best global perfusion is paramount [15]. Therefore, some centres advocate using bilateral axillary cannulation, as it maximises vertebral perfusion (posterior cerebral compartments) and reduces the risk of perioperative stroke [16,17]. Its benefit is more marked when a period of SACP over 40 min is expected, as there was no difference in the incidence of postoperative stroke and neurologic complication in shorter procedures [9]. Ideally, all patients undergoing aortic arch surgery would have a CT Cerebral angiography to assess the integrity of the Circle of Willis and a plan made to ensure bilateral hemispheric perfusion with monitoring of the cerebral saturation, as well as cerebrospinal fluid markers of hypoperfusion and ischaemia such as neuron-specific enolase and glial fibrillary astrocytic protein [16]. These diagnostic modalities are costly and are not readily available in most centres; there is also little evidence that these interventions would improve overall neurological outcomes. The importance of these markers and the overall impact of an incomplete Circle of Willis, as well as effective interventions, are unknown [16,18].

The coupling of cerebral flow and metabolism is essential for cerebral protection, and greater flow might increase the risk of cerebral oedema and embolic phenomena [15]. In porcine models, the optimal flow rate was between 6 and 15 mL/kg and flows higher than 15 mL/kg/min were associated with worse neuro-behavioural recovery [19]. The same is valid at the lower end of the spectrum, where perfusion pressures lower than 50 mmHg and flow rates less than 6 mL/kg/min were associated with signs of cellular cerebral ischaemia [20]. When multiple areas are perfused (i.e., bilateral axillary plus aortic root perfusion), it is vital to quantify the flow per area to avoid hyper- or hypoperfusion when changes in vascular resistance occur [21]. Maintaining a consistent (within 20% variation) cerebral oximetry percentage before and after SACP offers additional guidance to adjust the flow [15].

In addition to optimal cerebral perfusion, hypothermia is the second key component for organ protection. It was first employed back in 1953 by Lewis and Taufic, and the first use of hypothermic arrest for aortic surgery was achieved by Griepp and associates in 1975 [22,23]. The mechanisms of action have been widely explored. The goal is to reduce the metabolic rate and lower the demand for glucose to prevent adenosine triphosphate (ATP) depletion that leads to necrosis, to reduce free radical production, apoptosis and post-ischaemic cerebral oedema [24]. Moderate hypothermia causes burst suppression at 24.4 ± 4 °C and electrocerebral silence at deep hypothermia around 17.8 ± 4 °C, which minimises oxygen consumption and provides neuroprotection [25]. Based on the ratio of cerebral metabolic rates at temperatures of 10 degrees apart, estimates of the safe duration of hypothermic circulatory arrest at various temperatures were generated, showing that a safe period of deep hypothermic arrest at 14.1–20 °C is 21–33 min [25,26]. However, increasing evidence suggests that DHCA causes cerebral microvasculature endothelial dysfunction, worsens inflammatory response and increases neuronal damage, coagulopathy and acute kidney injury [27,28].

Moderate hypothermic circulatory arrest (MHCA) with SACP has been favoured in recent years [9,29]. Numerous single-centre trials have consistently shown that moderate hypothermia (24–28 °C) is associated with lower stroke rate, less bleeding and improved splanchnic organ recovery [30,31,32]. The benefit is more significant in patients with previous cardiac operations who underwent total aortic arch replacement [32]. However, even though using moderate hypothermia cerebral protection is considered safe and effective, there are concerns about the effect of the higher temperature range on the spinal cord during lower-body circulatory arrest [31].

The delivery and monitoring of regional perfusion and temperature management are macro approaches to organ protection. Various medical therapies, including anaesthetic agents, play a modest but growing role in the micro-protection of the brain. General anaesthesia with propofol, thiopental or volatile anaesthetic causes burst suppression on EEG, representing decreased or absent cerebral electrical activities [31]. They slow cerebral metabolism, potentiate GABA-mediated inhibition and alter cerebral blood flow. General anaesthetic with propofol inhibits free radical generation, promotes free radical scavenging and reduces eosinophilic apoptotic injury, which makes them attractive adjuvants to hypothermia and regional perfusion [32,33]. Corticosteroids have been widely used as pharmacological adjuvants to neuroprotective measures in thoracoabdominal surgery, but the evidence behind this is not very extensive [34,35]. Corticosteroids used in patients undergoing routine CPB have shown no benefit. However, there is no evidence related to its use in major aortic interventions [36,37]. Animal studies have shown benefits to preoperative steroid use, with attenuation of cerebral changes seen in DHCA, such as cerebral oedema and cerebral vascular leak, and have demonstrated better overall immunohistochemical profiles, but its translation to humans is yet unproven [38,39,40]. Novel molecular techniques that block endogenous miRNA regulator of peroxisome proliferator-activated receptors and protect the brain tissue during DHCA have been successfully tried in animal studies. Still, their use in humans has not been tested [41].

Lastly, preoperative planning, good team communication between surgery, anaesthesia and perfusion, and the cumulative experience of that trio are fundamental in ensuring the best organ protection.

## 3. Spinal Cord Protection for Aortic Arch Surgery

Patients undergoing aortic arch replacement are particularly vulnerable to spinal cord injury [42,43,44]. The combination of anatomical abnormalities in the descending aorta, the inevitable sublethal ischaemia during prosthesis implantation (lower body arrest) and the acute changes in the aortic perfusion following surgery pose a severe threat to spinal perfusion [41,45].

There is a significant variation in the overall incidence of perioperative spinal cord injury after a frozen elephant trunk (3 to 19%) [43,46]. The risk variation is associated with the anatomy of the aorta and the blood supply to the spinal cord. Humans have between one and three Adamkiewicz arteries; most people have three, and these are one component of a deeply interconnected collateral network [47,48]. Patients with poor development of the major radicular artery or defects in the anterior spinal arteries have an increased risk of paraplegia. Still, it is mostly the vast collateral network that sustains the blood supply when segments of the aorta are disabled, and its optimisation can impact neurological outcomes [49,50]. Patients with an intact descending aorta and a healthy collateral network are at low risk of spinal cord injury, and their incidence of paraplegia is between 1% and 5%, depending on the series (see Table 1) [3,51]. The intact collateral network is fed through the vertebral circulation, the lumbar plexus, splanchnic vessels and the subclavian artery, which provides sufficient blood supply to perfuse the spinal cord after the arch prosthesis is deployed [50]. In contrast, the high-risk group undergoes acutely radical changes in the flow dynamics after the frozen elephant trunk is deployed in the descending aorta, and these changes can lead to spinal cord ischaemia [52]. Patients at high risk of spinal cord injury (up to 5 to 15%) may include those with compromised major radicular arteries, a dissected descending aorta (type B-like) and those with a highly perfused posterior false lumen [52,53,54,55].

**Figure 1:**
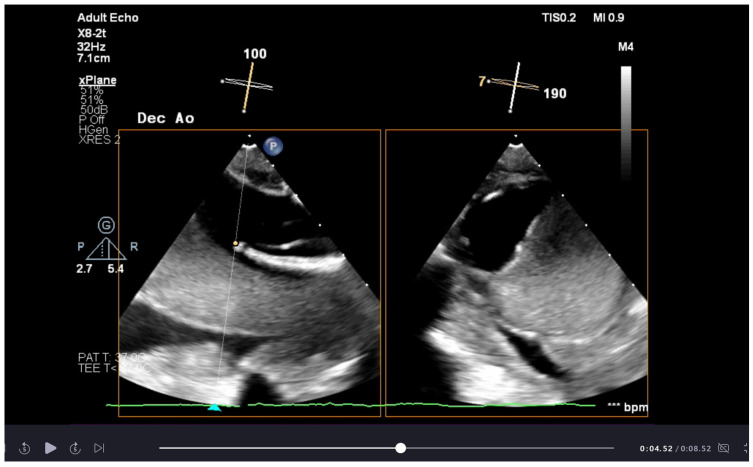
Transoesophageal echocardiogram image of thrombosis of false lumen post deployment of aortic graft.

After the elephant trunk deployment, the rapid stabilisation and exclusion of the false lumen due to thrombosis and occlusion in the first 24–48 h after the operation (see Figure 1) causes a progressive reduction in the spinal cord blood supply that can lead to delayed paraplegia [56,57,58]. As part of the postoperative adaption to this event, the damaged collateral network undergoes intensive angiogenesis within 24–48 h after the operation and ischaemic areas are revascularised [59]. There is early experience in spinal cord preconditioning through minimally invasive segmental artery coil embolisation, attempting to develop neo-vessels and improve spinal resilience before the operation [60,61]. Ultimately, the perioperative goal is to bridge to vascular recovery and maximise the flow in the existing vascular bed, ensuring a high perfusion pressure after surgery [56,61].

Some technical aspects of the surgery cannot be avoided, such as the period of lower-body ischaemia for the deployment and anastomosis of the frozen elephant trunk. The use of bilateral antegrade cerebral perfusion has been shown to provide perfusion to the spinal cord; both animal and human trials have shown that this is ineffective below the T8/T9 thoracic levels [58,62]. Hence, hypothermia still presents the best method of reducing irreversible spinal cord injury. Guidelines recommend the use of SACP plus a mild-to-moderate hypothermic approach of 25–28 °C either through systemic hypothermia on bypass or through regional hypothermia [59,60,61,63]. Deep hypothermia (18–22 °C) should be sought if lower-body ischaemia longer than 60 min is expected [30,62].

An additional strategy to facilitate cord perfusion is to reduce the pressure in the spinal compartment using cerebrospinal fluid (CSF) drainage with regional spinal cord hypotension [64,65]. Although the evidence for preventing intraoperative ischaemia using CSF drainage is limited, a few single-centre randomised trials have shown improved spinal cord functional recovery [66]. Some centres use CSF drainage routinely for all patients undergoing arch replacement, which may not outweigh the risk given that the spinal catheter insertion itself has a 1% risk of traumatic spinal injury, epidural bleeding and infection [66]. Other centres use it as a rescue technique after spinal shock is diagnosed. There are several limitations to this approach, given that patients cannot be assessed shortly after surgery because of the effects of anaesthesia and potential haemodynamic instability or bleeding. Perioperative coagulopathy is a contraindication for catheter insertion that can delay or impede its use as a rescue therapy. The authors favour a risk stratification approach for spinal cord injury, where patients that fall into the high-risk paraplegia group will have a CSF drainage before the operation and up to 48 h afterwards (see Table 1). Having the catheter inserted and patent allows immediate treatment of delayed paraplegia or spinal shock. For patients with a low risk of paraplegia, spinal catheter insertion can be reserved for those who develop neurological complications in intensive care.

Monitoring of spinal cord perfusion during aortic arch surgery presents significant challenges. We recommend monitoring femoral artery blood pressures in all patients who require LBCA. Femoral artery blood pressure is a good indicator of the restoration of blood flow to the true lumen after interposition of the graft [67]. However, femoral flow is insufficient to predict the presence of spinal cord ischaemia or the development of delayed paraplegia. Prolonged somatosensory evoked potentials can detect ischaemic changes in the case of delayed spinal cord ischaemia [67]. Despite that, it has limited usefulness during surgery due to factors such as inevitable lower body arrest, hypothermia and rewarming with delayed recovery of spinal cord function [68].

Pharmacological interventions for spinal cord protection have been employed, but no large trials to date show a significant effect of steroids for spinal cord protection. The use of high-dose corticosteroids is one of the strategies that is more utilised [69]. Animal models using high doses of corticosteroids prior to aortic cross-clamp significantly improves safe ischaemic time and lead to better neurological recovery [70]. Other similar studies have shown better perfusion profiles and neurological outcomes when applying steroids and CSF drainage compared to no intervention and steroids only [71]. The simultaneous use of steroids and other agents, such as mannitol or naloxone, is another recommended option with documented favourable results [72].

## 4. Additional Monitoring for Aortic Arch Surgery

Bilateral radial artery pressure monitoring is helpful during SACP and after subclavian reimplantation to ensure adequate flow as part of the spinal cord protection. Femoral artery pressure monitoring detects the blood flow in the descending aorta, although the prosthesis deployment needs to be confirmed with transoesophageal echocardiography (Videos S1 and S2). Transoesophageal echocardiography (TOE) is essential in the preoperative period in assessing the severity of the aortic pathology and for surgical planning, mainly when there is aortic root involvement. If a guidewire is used to direct the deployment of the elephant trunk prosthesis, TOE helps identify that the guidewire is in the true lumen. Once the prosthesis is deployed and the flow in the descending aorta is restored, TOE is used to confirm the correct deployment of the graft and assess the flow dynamic between the true and false lumen (Video S3) [73].

Transcranial Doppler (TCD) monitoring flow through large cerebral vessels (middle cerebral artery) [74] is helpful in the early detection of emboli and decreases blood flow in real time, especially in the postoperative period [75]. However, TCD requires significant training and is user-dependent, making its use challenging in the perioperative setting.

Genetic testing has been advocated in patients and their family members when they are diagnosed with an aortic aneurysm [76]. Mutations in the FBN1 gene are associated with rapidly increasing aortic size and aortic dissection at smaller diameters than currently recommended [76]. They are also known to have worse post outcomes during endovascular procedures. The 2022 ACC/AHA guidelines recommend that these patients have surgical intervention prior to achieving an aortic diameter of 5 cm to reduce the risk of severe postoperative complications [77]. The implications of gene therapy in improving the vascular matrix and reducing disease progression is worthy of further study.

## 5. Postoperative Management—Prevention and Treatment of Delayed SCI

Monitoring for neurological complications in the postoperative phase is an important part of aortic surgery care. While presentations may vary in terms of severity, from paraesthesia to single leg weakness or paraplegia, patients are often unable to express neurological symptoms due to ongoing sedation, encephalopathy, and delirium. Neurogenic shock with vasoplegia can also be masked by the presence of cardiogenic shock.

Sedation breaks are essential to performing a thorough neurological examination; however, strategies to minimise SCI must be applied regardless. Mean arterial pressure between 80 and 100 mmHg should be maintained while the patient is sedated unless the patient is actively bleeding, in which case coagulopathy should be actively treated to pursue those haemodynamic targets. CSF drainage should be continued in the intensive care unit to achieve a target pressure of 10–12 mmHg while patients are sedated. It can be increased to 12–15 mmHg once the patient is awake, and the neurological examination is normal. If the patient develops spinal shock, with sudden paraplegia and hypotension, the haemodynamic targets need to be increased (mean arterial pressure 100–140 mmhg) by any means, including the use of vasoconstrictors such as noradrenaline or vasopressin. Naloxone has been used as a second-line pharmacological agent to reverse delayed spinal cord injury [78]. Its role is to reduce the quantity of the excitatory amino acids in cerebrospinal fluid, which accumulates in periods of spinal cord ischaemia and subsequently have a neurotoxic effect [79]. Animal studies have shown that endogenous opioids may reduce microcirculatory blood flow and that naloxone may indeed improve perfusion in neurological ischaemic injury. Hence, a rescue attempt with naloxone bolus followed by an infusion can be trialled [80]. Adjuvant monitoring, such as utilising evoked potentials (MEPs and SSEPs), has been proposed to predict immediate and delayed SCI. Still, their sensitivity and specificity in patients undergoing aortic arch replacement are low [81,82].

## 6. Conclusions

Protection of the brain and spinal cord is essential when managing patients undergoing surgery of the aortic arch. The patient’s risk assessment to determine severe neurological complications is essential, and a multidisciplinary perioperative plan should be tailored to tackle the risk. Prevention of ischaemia by increasing end-organ perfusion and by employing techniques to improve their resilience should be at the core of the strategy. Moderate hypothermia and bilateral antegrade cerebral perfusion are the preferred methods to manage the sometimes conflicting requirements to maintain both spinal cord and cerebral perfusion. Postoperative care should focus on the prevention of delayed paraplegia and its early detection and treatment when it happens.

## Figures and Tables

**Table 1:** Factors associated with risk of neurological complications after aortic arch surgery.

	High Risk	Low Risk
Demographics	Age > 70	Age < 50
	Hx of CKD	Low arterial calcification score
	Hx of diabetes mellitus Redo cardiovascular surgery Previous stroke Atherosclerotic disease of aorta	
Factors Associated with Pathology	Type A aortic dissection with carotid involvement	Aortic aneurysm with no dissection
	Type B aortic dissection with posterior false lumen Highly perfused false lumen Aneurysm w/abdominal aortic distention	Poorly perfused false lumen Platelet count > 60 × 109 L
Factors Associated with Procedure	Femoral artery cannulation	Subclavian graft
	Prolonged LBCA > 50 min	Dual cannulation
	Lack of subclavian artery reimplantation	
	Postoperative hypotension	

## Data Availability

This is a review article of current practices, there was no collection of patient data. Images used have been anonymised for privacy and ethical reasons.

## Supplementary Materials

The following supporting information can be downloaded at: https://www.mdpi.com/article/10.3390/jcdd12040130/s1, Video S1: Transoesophageal echocardiogram showing thrombosis of the false lumen post deployment of aortic graft. Video S2: Transoesophageal echocardiogram showing aortic dissection of the descending lumen with blood flow through true lumen. Video S3: Transoesophageal echocardiogram showing blood flow the deployed graft.
